# Expression of Wheat High Molecular Weight Glutenin Subunit 1Bx Is Affected by Large Insertions and Deletions Located in the Upstream Flanking Sequences

**DOI:** 10.1371/journal.pone.0105363

**Published:** 2014-08-18

**Authors:** Yuke Geng, Binshuang Pang, Chenyang Hao, Saijun Tang, Xueyong Zhang, Tian Li

**Affiliations:** 1 Key Laboratory of Crop Gene Resources and Germplasm Enhancement, Ministry of Agriculture/Institute of Crop Science, Chinese Academy of Agricultural Sciences, Beijing, China; 2 College of Biological sciences, China Agricultural University, Beijing, China; 3 Beijing Engineering and Technique Research Center of Hybrid Wheat, Beijing Academy of Agricultural and Forestry Sciences, Beijing, China; Instituto Valenciano De Investigaciones Agrarias, Spain

## Abstract

To better understand the transcriptional regulation of high molecular weight glutenin subunit (HMW-GS) expression, we isolated four *Glu-1Bx* promoters from six wheat cultivars exhibiting diverse protein expression levels. The activities of the diverse *Glu-1Bx* promoters were tested and compared with β-glucuronidase (*GUS*) reporter fusions. Although all the full-length *Glu-1Bx* promoters showed endosperm-specific activities, the strongest GUS activity was observed with the *1Bx7^OE^* promoter in both transient expression assays and stable transgenic rice lines. A 43 bp insertion in the *1Bx7^OE^* promoter, which is absent in the *1Bx7* promoter, led to enhanced expression. Analysis of promoter deletion constructs confirmed that a 185 bp MITE (miniature inverted-repeat transposable element) in the *1Bx14* promoter had a weak positive effect on *Glu-1Bx* expression, and a 54 bp deletion in the *1Bx13* promoter reduced endosperm-specific activity. To investigate the effect of the 43 bp insertion in the *1Bx7^OE^* promoter, a functional marker was developed to screen 505 Chinese varieties and 160 European varieties, and only 1Bx7-type varieties harboring the 43 bp insertion in their promoters showed similar overexpression patterns. Hence, the *1Bx7^OE^* promoter should be important tool in crop genetic engineering as well as in molecular assisted breeding.

## Introduction

Hexaploid wheat (*Triticum aestivum* L.) is one of the most important human food sources. Its complex genetic background leads to great diversity in nutritional and processing qualities among cultivars. High molecular weight glutenin subunits (HMW-GSs) are the main grain storage proteins in the endosperms of wheat and related species [1], [2]. Although HWM-GSs grain storage proteins account for only about 12% of the total protein [3], they play a key role in wheat gluten as the skeletal network that to a large extent determines its structure and formation [4]. The compositions and quantities of allelic variation in HMW-GS genes substantially affect the taste and appearance of dough products, such as Chinese noodles and European bread [5]. Therefore, improvement of flour quality based on superior HWM-GS alleles is necessary to meet changing consumer demands.

Both qualitative and quantitative effects of HMW-GS subunits are important for flour quality [6], [7]. In the process of breeding, high dough strength is used as a predictor of good-quality bread wheat; and overexpression of Glu-1Bx7 by way of allele *1Bx7^OE^* makes an important contribution to high dough strength in some cultivars [8], [9]. Expression of HMW-GS is regulated by three major factors, which are at the genomic level (gene duplication), transcriptional level and translational level [10]–[13]. Transcriptional regulation driven by *Glu-1* 5′-upstream flanking regions might provide strategies for improving grain quality in wheat breeding programs [14]. A number of crucial *cis*-acting elements from HMW-GS promoters of various wheat cultivars have been investigated and characterized, as these could affect tissue specificity or expression activity, including conservative endosperm-specific motifs, such as the GCN4 motif [15], the prolamin box [16], AACA/TA motif [17], RY repeat motif [18], and Skn-1 [19], [20], each of which is capable of exerting temporal expression [21], [22]. However, the basis of transcriptional regulation of divergence caused by large insertion and deletion (InDel) alterations in HMW-GS promoter regions is still not clear. As reported earlier, a tandem 54 bp duplication, known as the “cereal-box” located at −400 bp in the *1Bx* promoter may enhance endosperm-specific expression [1], suggesting this duplicated region might be a key region for control of gene expression [23], [24]. There is a 185 bp MITE insertion in the promoters of *1Bx14* and *1Bx20*, but functional verification indicated that this insertion had little effect on gene expression [25]–[27]. A 43 bp insertion found at −1000 bp in the *1Bx7^OE^* promoter was significantly associated with the overexpression phenotype. It was speculated that the overexpression was brought about by gene duplication mediated by the insertion of a retroelement [13], and there was no further study concerning the 43 bp InDel effect on protein expression. Therefore, more experimental data are needed to clarify the effect of InDels in HMW-GS promoters.

Highly active endosperm-specific promoters serve as an important genetic resource for high-quality and high-yield wheat breeding. Use of seed storage protein gene promoters is an attractive strategy for obtaining target gene products exclusively from crop kernels. A number of seed-specific promoters from barley, rice, maize and other species have been investigated functionally [15], [21], [28]. Transgenic crops with favorable gene stacking require different tissue-specific promoters from various cereals, as this is helpful to reduce homology-based transcriptional gene silencing [29], [30]. HMW-GS promoters from wheat, although containing endosperm-specific motifs, may not be spatially controlled in the same way as in their original genetic backgrounds due to subtle differences in respective regulation systems [31]. Hence, further research of key motifs from tissue-specific promoters would boost applicability in genetic engineering.

Among hexaploid wheat HMW-GSs, Glu-1Bx often shows the highest level of expression [32]. We therefore set out to analyse *1Bx* promoter sequence characteristics to uncover the transcriptional regulation mechanism. Based on diverse protein expression levels in six wheat cultivars, we isolated four *Glu-1Bx* promoters in approximately 2.2 kb of length and further validated their functions. By comparison with these upstream sequences, several large InDels such as a 43 bp InDel, a 54 bp duplication and a 185 bp MITE resulted in major divergences among the four promoters, including the *1Bx7* promoter (*Pro*-*1Bx7*), *1Bx7^OE^* promoter (*Pro*-*1Bx7^OE^*), *1Bx13* promoter (*Pro*-*1Bx13*) and *1Bx14* promoter (*Pro*-*1Bx14*). The promoter sequence variation was shown to be an important factor causing differential expression in transient expression systems and in transgenic rice plant assays. Notably, *Pro*-*1Bx7^OE^* is a highly active endosperm-specific promoter that can be made available for crop improvement by transgenic methods. Moreover, we developed a new specific molecular marker in terms of the 43 bp insertion residing in the *1Bx7^OE^* promoter, with which we screened 505 Chinese and 160 European cultivars [33]. We found that this functional marker is significantly associated with *1Bx7* overexpression. Our results further showed that transcriptional regulation might be responsible for *1Bx* expression diversity to a larger extent than initially expected.

## Materials and Methods

### Plant materials

Hexaploid wheat (*Triticum aestivum* L.) cultivars (cv.) Yanzhan 1, Atlas 66, Jimai 20, Xiaoyan 54, Yunmai 33 and Chinese Spring were grown in the field. Endosperm of Xiaoyan 54 was prepared for transient expression assays, and rice (*Oryza sativa* L. ssp *japonica*) cv. Kita-ake was used to produce stable transformants. Materials used for molecular marker screening included 505 Chinese and 160 European cultivars [33].

### SDS-PAGE and quantification of HMW-GSs

Protein fractions were extracted from single wheat kernels using a previously reported HMW-GS extraction protocol [34]. Identical amounts of protein extracted from seeds of different varieties were separated by SDS-PAGE and visualized by Coomassie Blue staining as described by Zhang et al. [35]. Densitometric analyses of 1Bx subunits were carried out by Quantity One software (Bio-rad, USA). The value of the optical density multiplication area was used to quantify HMW-GS expression.

### Promoter isolation and cis-element prediction

DNA extraction was performed as previously described [36]. Using a pair of specific primer sets, 1Bx2258F/R (Table S1), four full-length *1Bx* promoters, ∼2.3 kb in size, from six wheat varieties were isolated, gel purified and sequenced. Putative regulatory elements within the *1Bx* promoter were predicted using the Plant Cis-acting Regulatory DNA elements (PLACE) database [37] combined with a previous report [38].

### Construction of promoter-GUS chimeric genes and subsequent transformation

Several full-length and truncated *1Bx* promoters were obtained by PCR amplification with primers introducing DNA restriction enzyme sites for convenient subcloning (Table S1), and cloned into a modified vector PAHC25 [39] for transient expression, and then subcloned into binary vector pCAMBIA1391z, containing the reporter gene *GUS* under the control of different *1Bx* promoters. In transient expression experiments, immature embryos harvested at 12–14 days post anthesis (DPA) were used for bombardment as described by Ortiz et al. [40]. Different *1Bx* promoter-*GUS* constructs were tested in transient expression assays as described previously [41]. For stable transformation, the binary vector constructs were first introduced into *Agrobacterium tumefaciens* strain EHA105, and then rice transformation was carried out as described by Cho et al. [42]. Transgenic rice plants were selected on medium containing 50 mg L^−1^ of hygromycin, and positive lines were grown in the field for further analysis.

### PCR and Southern blot analyses of transformed rice plants

Genomic DNA was isolated from leaf tissues of transformed rice plants as previously described [36]. PCR analysis for molecular identification of transgenic rice plants was performed using a set of specific primers for the *1Bx* promoter and *GUS* gene (listed in Table S1).

For Southern blot analysis, genomic DNA (10 µg) from different transgenic lines were digested with *Bam*HI or *Hind*III (New England Biolabs, USA). Digested DNA was separated by electrophoresis in 0.8% (w/v) agarose gels, and then transferred to Hybond-N^+^ membranes (Amersham Biosciences, USA) and hybridized with a *GUS* gene fragment labeled with [α-^32^P]dCTP as described previously [36].

### Histochemical GUS assay

Wheat endosperms undergoing transient expression and different tissues of T_3_ transgenic rice were used for histochemical GUS assays. GUS staining was performed as described by Kosugi et al. [43]. Images of stained samples were captured using an MZ16 High-tech Stereomicroscope (Leica, Germany). Stained GUS spots were counted, and for statistical comparison, data of each sample was expressed as the mean number of blue spots per endosperm.

### Quantification of expression of the *GUS* gene under control of *1Bx* promoters in transgenic rice plants

Total RNA was extracted from kernels of 3 independent positive T_3_ transgenic rice lines at 10–18 DPA. Transcriptional levels of *GUS* in all stable transgenic lines were quantified by quantitative real-time PCR (qRT-PCR) with a 7300 Real-time PCR system (Applied Biosystems, USA) using Power SYBR Green PCR Master Mix (Applied Biosystems, USA). Details of primer pairs used for qRT-PCR are given in Table S1. The specificity of the primer sets was assured by confirmation that the resulting products appeared as single peaks in real-time melting temperature curves and as single fragments after separation by agarose gel electrophoresis. To confirm adequate amplification PCR efficiency was assessed using a sample dilution series as templates [44]. Amplification plots and predicted threshold cycle values were obtained from three independent biological replicates with SDS software version 2.1 (Applied Biosystems, USA). *GUS* gene expression levels were presented as fold-changes calculated using the comparative threshold cycle (CT) method as described [45] with rice *GAPDH* used as the internal control.

## Results

### Identification of HMW-GSs by SDS-PAGE

**Figure 1:**
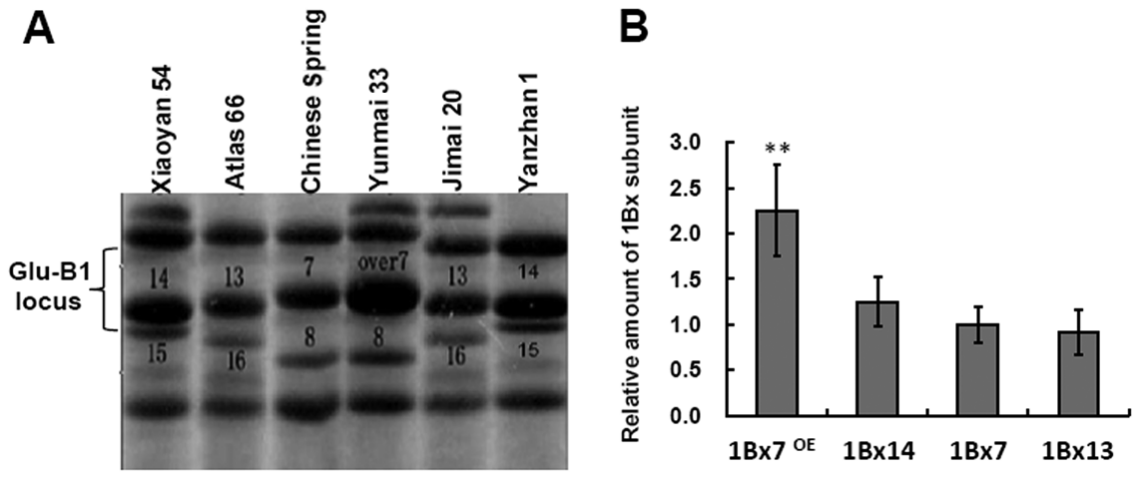
HMW-GSs from six common wheat cultivars. (A) SDS-PAGE profiles of HMW-GSs. 1Bx7 (Chinese Spring), 1Bx13 (Atlas 66 and Jimai 20), 1Bx14 (Xiaoyan 54 and Yanzhan 1) and 1Bx7^OE^ (Yunmai 33). (B) Relative amounts of four 1Bx subunits estimated by densitometric analysis.

HMW-GSs of six wheat cultivars were separated by SDS-PAGE (Figure 1A). Their subunit compositions varied from each other (Table S2). Evidently, expression levels of Glu-1A and Glu-1D are generally lower than Glu-1B. There were four allelic variants of *1Bx* among the six cultivars, namely *1Bx7^OE^, 1Bx14, 1Bx13* and *1Bx7*. The protein level of 1Bx7^OE^ in Yunmai 33 was much higher than that of 1Bx in other cultivars, about 2.2-fold that of 1Bx13 and 1Bx7 and 1.8-fold that of 1Bx14 (Figure 1B).

### Comparative analysis of upstream sequences of *Glu-1Bx*

**Figure 2:**
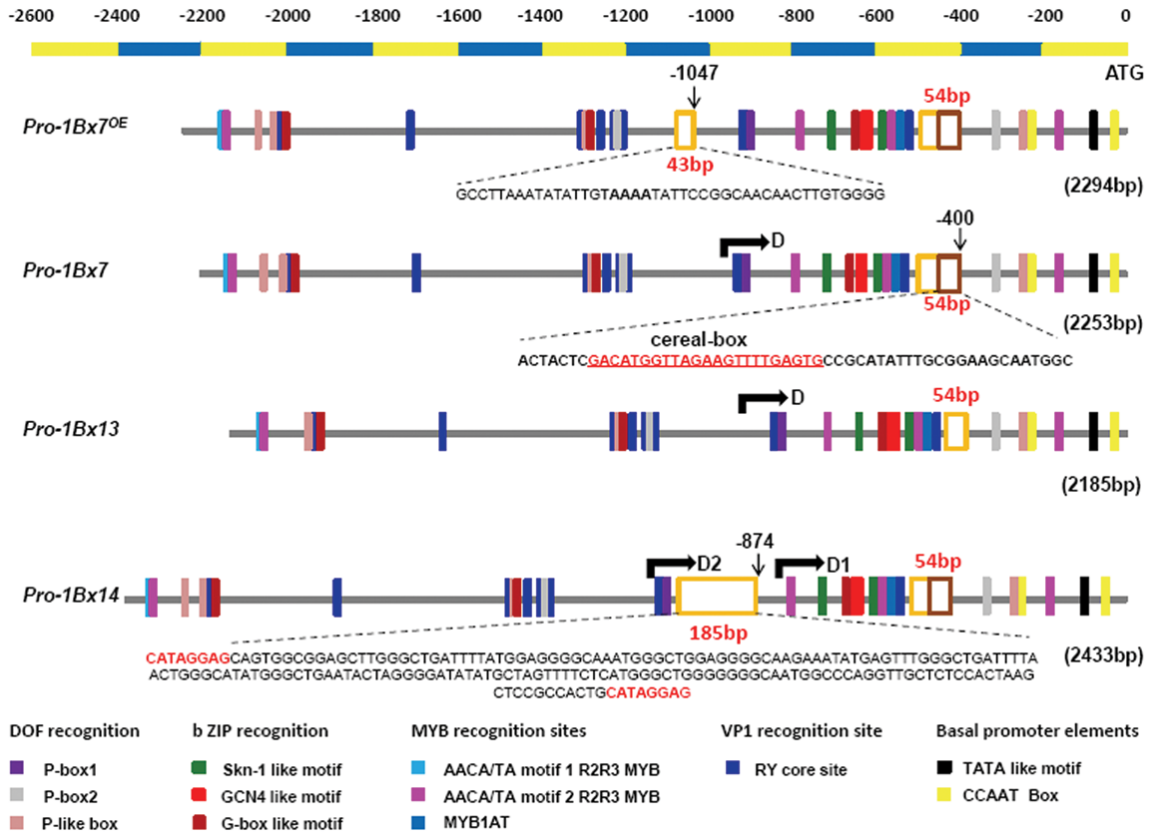
Schematic structure of four *1Bx* promoters. Regulatory elements are indicated by colored rectangles. InDels are labeled with hollow boxes and core sequences are listed in detail under the sketch map. The red underlined sequence shows the “cereal box” in the 54 bp duplication. The 8 bp bases in red at both ends indicate the target site duplication (TSD) of the MITE. Positions of upstream primers used for obtaining truncated promoters are indicated with black arrows.

Four types of 5' flanking sequences of *1Bx* alleles were isolated with the specific primer pair, 1Bx2258F/R (Table S1 and Figure S1). They were 2,294, 2,253, 2,185 and 2,433 bp in length for *Pro*-*1Bx7^OE^*, *Pro*-*1Bx7*, *Pro*-*1Bx13*, and *Pro*-*1Bx14*, respectively. The four 5' proximal flanking regions contained five common motifs, including DOF recognition sites, bZIP recognition sites, MYB recognition sites, VP1 recognition sites and basal promoter elements (Figure 2 and Table S3), which are conserved in promoters of genes that encode most seed storage proteins [38]. In addition to several single-base substitutions or small deletions, the presence of sequence insertions or deletions (InDels) constituted the main differences among the four entire promoter regions (Figure S2). By comparison with the *1Bx7* promoter, the *1Bx13* promoter has a 54 bp deletion at −400 upstream from the start codon (54 bp duplication position), the *1Bx14* promoter contains a 185 bp MITE insertion at −874, consistent with previous reports [26], [46], and the *1Bx*7*^OE^* promoter possesses a 43 bp insertion at −1047, which is always associated with the overexpression phenotype [12].

### The transient expression results for different *1Bx* promoters

**Figure 3:**
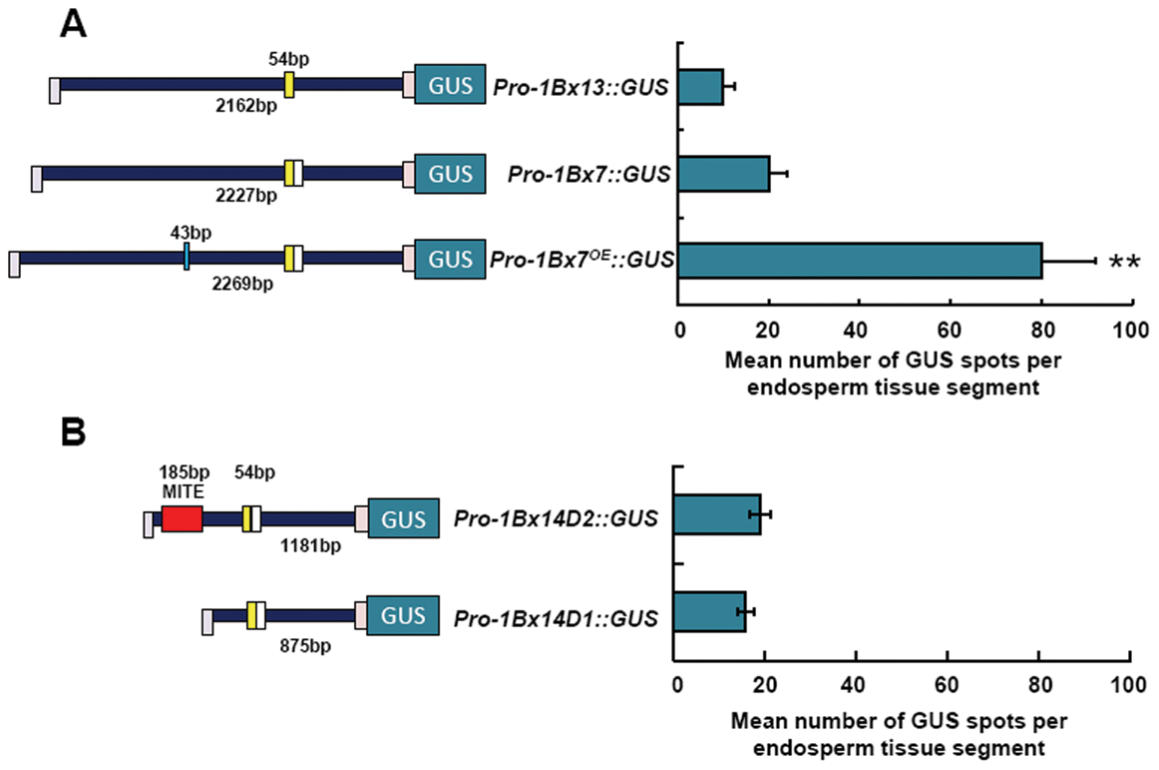
Schematic representation of constructs used for transient expression assays in wheat endosperms and *GUS* activities driven by full-length and truncated *1Bx* promoters. (A) Effects of full-length *1Bx7*, *1Bx13* and *1Bx7^OE^* promoters on transient expression. (B) Effect of the 185 bp MITE from the *1Bx14* promoter on transient expression. **P<0.01 (student’s *t*-test) indicates significant difference from others.

We compared the expression efficiencies of the full-length promoters of *1Bx7*, *1Bx13* and *1Bx7^OE^* by means of transient expression assays in wheat endosperms (Figure S3). and *GUS* driven by the *1Bx*7*^OE^* promoter exhibited much higher activity than when driven by the *1Bx7* and *1Bx13* promoters (Figure 3A). Since the 43 bp InDel represents the difference between the *1Bx7* and *1Bx*7*^OE^* promoter sequences, we speculated that the 43 bp insertion enhanced the endosperm-specific expression. In addition, the *1Bx13* promoter activity was lower than that of the *1Bx7* promoter, which lacks the 54 bp duplication present in the *1Bx13* promoter. We further investigated the effect of the 185 bp MITE on *1Bx14* expression. Two truncated *1Bx14* promoters were fused to *GUS*, and transient expression results showed that *GUS* expression driven by a 1,192 bp *Pro-1Bx14-D2* was slightly higher than that driven by the 873 bp *Pro-1Bx14-D1* (Figure 3B), confirming that the MITE might positively but weakly affected transcription of *1Bx14* [26].

### Histochemical and quantitative assays in transgenic rice

**Figure 4:**
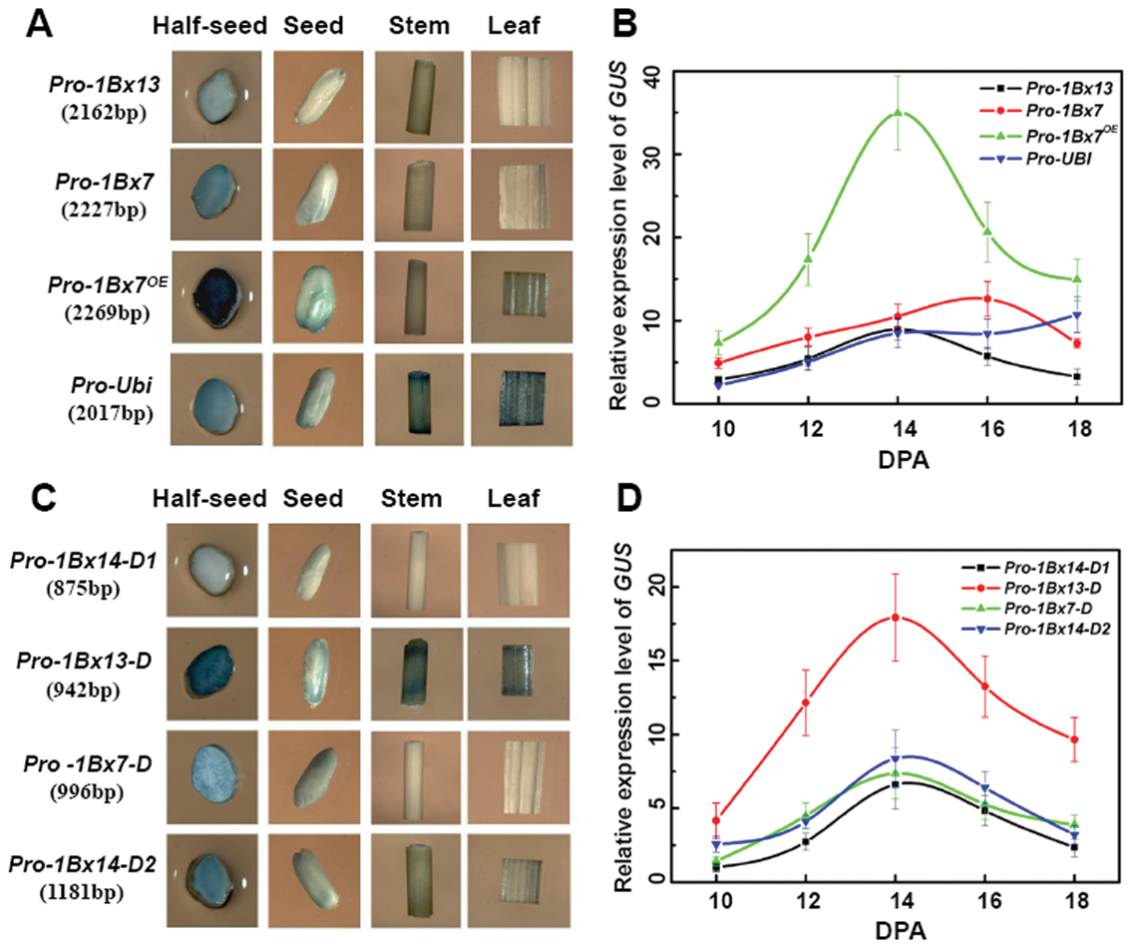
GUS staining of various tissues and quantitative analysis in developing endosperm of transgenic rice. (A) and (C) Histochemical analysis of *GUS* driven by different promoters in transgenic rice tissues collected at 15 DPA; (B) and (D) Relative expression levels of *GUS* in seeds from different transgenic lines during 10 to 18 DPA based on qRT-PCR. Rice *GAPDH* was used as the internal control. Values are shown as means ± s.d (standard deviation) of three independent experiments and three biological replicates. Colored lines at the top right corner represent different transgenic plants.

Chimeras were constructed using different *1Bx* promoters fused to *GUS* and then transformed into rice. Through GUS staining assays in the T_3_ generation of stable transgenic plants, we detected *GUS* activity driven by the three full-length *1Bx* promoters only in the seeds and not in stems or leaves collected at 15 DPA. These results indicated that the promoters are endosperm-specific (Figure 4A). In contrast, GUS staining was observed in all tissues of transgenic rice carrying the *Ubiquitin* promoter-*GUS* construct (Figure 4A). Therefore, the full-length *1Bx* promoters contained necessary *cis*-elements that specify endosperm-specific regulation in both wheat and rice. Consistent with the transient expression results, the full-length *1Bx7* ***^OE^*** promoter with the 43 bp insertion exhibited much higher *GUS* activity than either the full-length *1Bx7* or *1Bx13* promoters (Figure 4A). Southern blot analysis confirmed that the transgenic rice lines had single copies of the *GUS* gene (Figure S4); therefore the comparative results of promoter activities were convincing.

We also investigated the activities of different truncated *1Bx* promoters in transgenic rice. Except for the *Pro-1Bx13-D* promoter, other truncated promoters, including *Pro-1Bx14-D1/D2* and *Pro-1Bx7-D*, retained endosperm-specific expression activity (Figure 4C). Absence of the 54 bp duplication led to loss of endosperm-specific expression controlled by the *Pro-1Bx13-D* promoter, thus indicating that the fragment removed from the *1Bx13* promoter (−943∼−2162) contained necessary *cis*-elements that restricted expression to the endosperm. Like transient expression results, *GUS* expression directed by *Pro-1Bx14-D2* harboring the 185 bp MITE was higher than that directed by *Pro-1Bx14-D1*.

To confirm the results of GUS staining in seeds of transgenic rice, we applied qRT-PCR to determine expression levels of *GUS*. *GUS* expression detected in seeds during 10–18 DPA showed that expression driven by *1Bx* promoters increased rapidly from 10 DPA to 14 DPA, and reached a peak level at 14–16 DPA (Figure 4B and 4D). As the control, *GUS* expression driven by the *Ubiquitin* promoter maintained a relatively constant level through 10 to 18 DPA (Figure 4B). The results of *GUS* expression at 14–16 DPA were highly consistent with those of GUS activities based on histochemical staining (Figure 4A and 4C), confirming that the protein expression pattern was similar to the gene expression pattern at the mRNA level.

### Phylogenetic analysis of HMW-GS promoters

**Figure 5:**
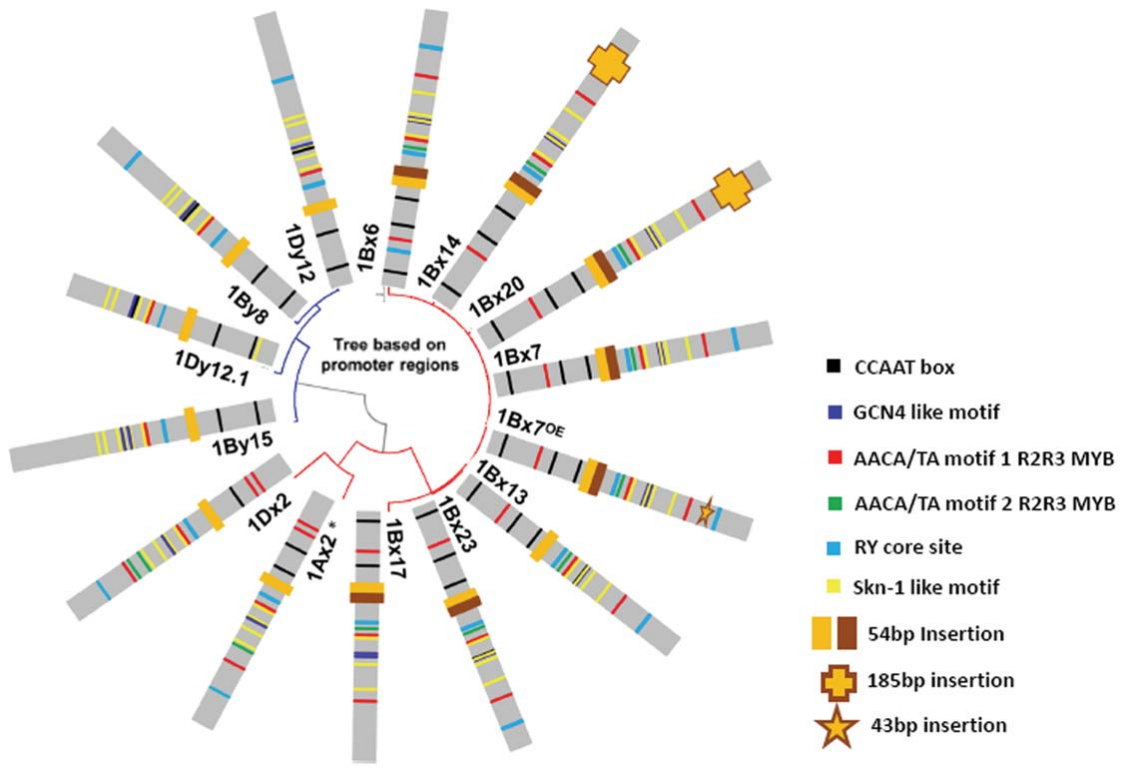
Phylogenetic analysis of 14 *HMW-GS* promoters. Neighbor-Joining Tree of partial length (-1 ∼ -1200 bp upstream of the start codon) sequences of *Glu-1* from *Triticum aestivum* L. and other wheat-related grass species. This work was done with the MEGA program (Version 5.2). InDels and conserved cis-elements are shown by markers in different colors.

To address the question of whether these InDels are present in other *HMW-GS* promoters, we analyzed promoters from 14 different wheat *HMW-GS* genes [1], [26], [47] and identified the regulatory motifs related to endosperm-specific expression in the promoter regions about 1,200 bp upstream of the initiation codon. The numbers of regulatory motifs obviously differed among the different HMW-GS promoter sequences (Figure 5). In phylogenetic analysis, all *Glu-1Bx* promoters clustered together in one branch. The 185 bp MITE insertion was present in both the *Glu-1Bx14* and *Glu-1Bx20* promoters. The 54 bp tandem duplication was absent in non*-Glu-1Bx* promoters, but present in all *Glu-1Bx* promoters except the *Glu-1Bx13* promoter. Therefore, InDels contributed to the diversity in HMW-GS promoters, which is an important means for evolution of HMW-GS genes. These large fragment InDels can be used as a potential resource for creating new alleles.

### Development of a molecular marker for the 43 bp insertion and its distribution in natural populations

**Figure 6:**
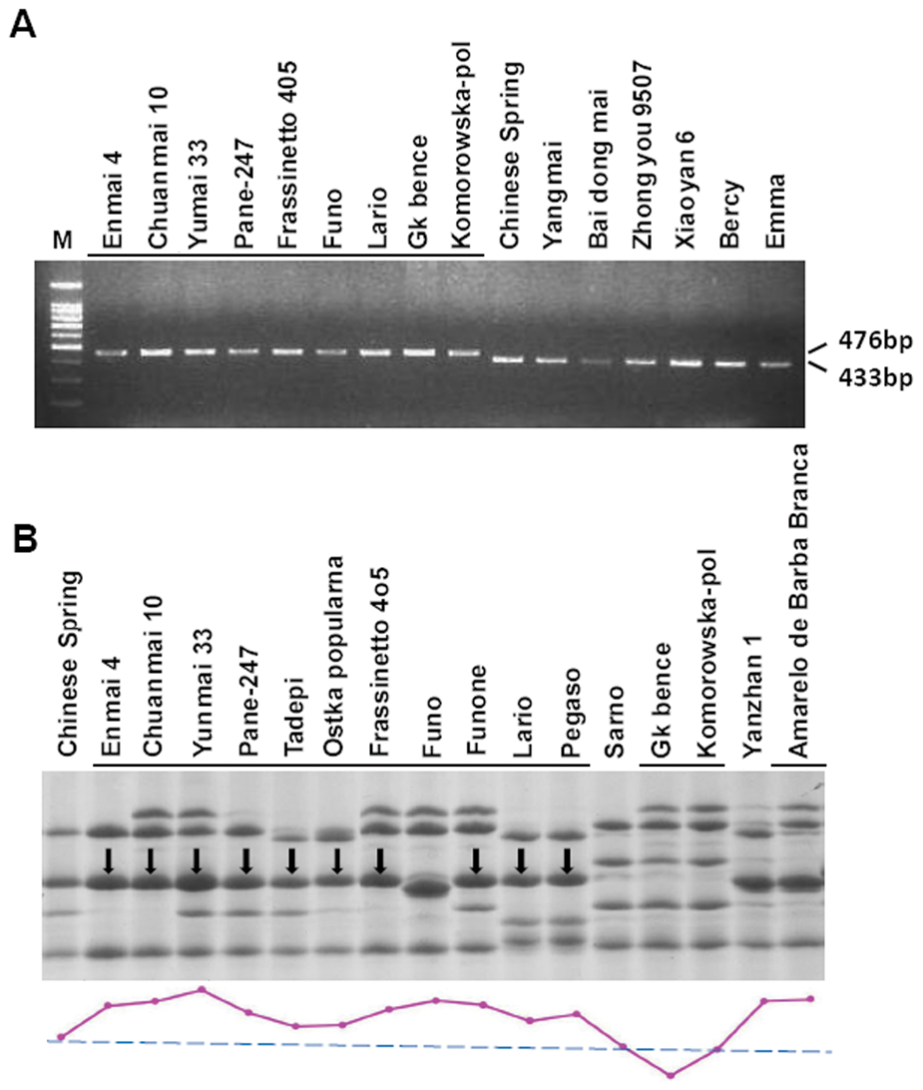
Electrophoretic separation of PCR products from *1Bx* promoters with or without the 43 bp insertion, and SDS-PAGE profiles of HMW-GS of wheat cultivars with the insertion. (A) PCR assays for *1Bx* promoters on a 2% agarose gel. M: 100 bp DNA Ladder. Underlined accessions possess the 43 bp insertion. (B) SDS-PAGE assay of HMW-GS from different accessions containing the 43 bp insertion. Underlined accessions possess the 43 bp insertion. Down black arrows indicate *1Bx7* with 43 bp insertion in accessions from China and Europe. Chinese Spring, Yanzhan 1 and Samo were used as controls. The purple curve represents the relative amounts from different 1Bx subunits.

Based on the 43 bp insertion sequence in the *1Bx7^OE^* promoter, we developed a new molecular marker that differed from those previously reported [34]. Our marker can precisely identify the insertion in HWM-GS promoters among common wheat varieties. PCR amplification resulted in two kinds of bands that distinguish promoters with the 43 bp insertion (a 476 bp fragment) from those without (a 433 bp fragment) (Figure 6A). Among 505 Chinese and 160 European accessions surveyed, we found 3 Chinese and 11 European varieties with the 43 bp insertion when we used this marker. HMW-GS profiles of accessions containing the 476 bp marker were later obtained by SDS-PAGE (Figure 6B). The presence of particular 1Bx alleles was determined by densitometric analysis; 10 accessions (3 from China and 7 from Europe) exhibited a 1Bx7 overexpression phenotype relative to Chinese Spring used as a control (Figure 6B and Table S4). We also identified some types of *1Bx6* and *1Bx14* with the 43 bp insertion (Figure 6B and Table S4), and confirmed its presence by DNA sequencing. However, these two types of 1Bx did not show overexpression at the protein level. Therefore, the 43 bp insertion in promoters preferentially enhanced 1Bx7 expression although no obvious differences were found among the upstream regions of *1Bx6*, *1Bx14* and *1Bx7^OE^*.

## Discussion

HMW-GSs represent a set of important seed-storage proteins, and both their composition and quantity significantly affect wheat flour quality [7]. Since gene transcriptional regulation is the dominant means of control in production of proteins [48], we isolated four promoter sequences of *Glu-1Bx* and investigated their effects on gene expression. Although the reporter gene driven by all the *1Bx* promoters exhibited an endosperm-specific expression pattern, the *1Bx7^OE^* promoter from cv. Yunmai 33 produced a markedly stronger activity than other promoters. Previous studies showed that gene duplication was the cause of 1Bx7 overexpression in some wheat cultivars [49]. The connection between the strong activity of the *1Bx7^OE^* promoter and high protein level produced by the 1Bx7^OE^ subunit clearly indicated that transcription regulation is also a factor in 1Bx7 overexpression. We therefore concluded that multiple factors, including gene duplication and transcriptional regulation determine the expression of *1Bx7*. This work revealed a complex regulatory network of HMW-GS expression in wheat.

### The 43 bp insertion at −1047 bp is closely associated with high expression of 1Bx7^OE^

Large InDels in promoter regions often result in higher rates of transcriptional divergence [50]. In this study, we identified a 43 bp InDel, a 54 bp duplication and a 185 bp MITE in different *Glu*-*1Bx* promoters, and they accounted for the main differences among *1Bx* promoter sequences. These InDels affected the expression levels of the genes. The presence of the 43 bp insertion at position −1047 upstream of the start codon was shown to be closely associated with high expression levels of the 1Bx7^OE^ subunit [12], [13]. We verified that the 43 bp insertion can serve as a strong enhancer to improve expression of the gene by comparing the transcriptional activities between full-length *1Bx7* and *1Bx7^OE^* promoters (Figure 3A; Figure 4A and 4B). Since there are no known *cis*-elements in the 43 bp insertion, this insertion may facilitate evolutionary tuning of gene expression by affecting local chromatin structure and nucleosome positioning [50].

### The 185 bp MITE insertion at −874 bp of *1Bx14* might slightly affect transcription

The 185 bp MITE insertion located at −874 bp in the *1Bx14* promoter may be a remnant of an earlier transposition of a large element or of small, highly repeated elements [51]. In the present study, the *1Bx14* promoter with or without the 185 bp MITE, did not produce a significantly different activity in the transient system or in stable transgenic rice assays, suggesting that it might only slightly affect the transcriptional regulation (Figure 3B; Figure 4C and 4D). The 185 bp MITE exists in both hexaploid and tetraploid wheat, and may be linked to the polyploidization event affecting the constitutions and activities of the genomes of grass species [26].

### A 54 bp cereal-box motif is necessary for endosperm-specific expression

The tandem 54 bp duplication at position −400 contains the “cereal-box” implicated in seed-specific expression [1]. Our data demonstrated that the *1Bx13* full-length promoter harboring one 54 bp deletion retains endosperm-specific activity, but a *1Bx13* promoter truncated at −942 bp lacks endosperm-specificity accompanied by increased activity (Figure 4C). We speculate that the 54 bp deletion might complement essential *cis*-elements in the region −940 to −2000 bp of the *1Bx13* promoter to effectively control gene endosperm-specific expression. Without the aid of the *cis*-elements in the region, only one 54 bp cereal-box motif may not be enough to restrict gene expression to endosperm. Based on phylogenetic analysis of HWM-GS promoters, only the *1Bx13* promoter and non-*1Bx* promoters contain a 54 bp deletion (Figure 5). This tandem 54 bp duplication must have occurred before hexaploidization because it is also present in tetraploid wheat. Flanking-sequence divergence was also noted from extensive DNA sequencing analysis of a-gliadin genes [52]. The basis of HMW-GS evolution is repeated sequence events that lead to new alleles [53].

### A simple PCR marker was developed to target high expression of 1Bx7 and 1Bx7^OE^

Since previous 43 bp InDel marker covers a region of 1.2∼1.3 kb that also contains other InDels such as the 185 bp MITE and 54 bp duplication [34], a new specific marker based on the 43 bp insertion was developed and used effectively in two independent wheat populations combined with SDS-PAGE electrophoresis analysis to identify 1Bx7 overexpressing cultivars. Interestingly, the 43 bp insertion exists not only in the *1Bx7* promoter but also in other *1Bx* promoters such as those of *1Bx14* and *1Bx6* (Figure 6B). Despite harboring the 43 bp insertion in the promoters, the 1Bx14 and 1Bx6 subunits produce no significant increases in protein compared to subunit alleles without the insertion (Figure 6B). The likely reason is that a co-regulatory factor linking the 43 bp insertion to expression efficiency is present in the 1Bx7 alleles or regulation at the translational level might strongly influence the divergence in expression between 1Bx7 and non-1Bx7 subunits.

### Putative additive effects of gene duplication and transcriptional regulation on 1Bx7 expression

According to the literature, it is concluded that the 1Bx7 overexpression phenotype is mediated by an LTR retroelement resulting in gene duplication along with the polyploidization event [13]. In the present study, we confirmed that a 43 bp insertion situated in the *1Bx7^OE^* promoter is capable of strengthening transcriptional activity markedly through transient expression and transgenic rice assays. By using molecular markers which can be used to indicate *1Bx7* gene duplication [13], we found that only the cultivar Yunmai 33 has both the 43 bp InDel and two 1Bx7 copies (gene duplication), while other 9 cultivars with the 43 bp InDel have only one 1Bx7 copy (Table S4). Although the 1Bx7 subunit of Yunmai 33 is the most abundant in this study (Figure 6B), other cultivars with the 43 bp InDel demonstrate higher 1Bx7 expression than the control, especially Chinese cultivars Enmai 4 and Chuanmai 10 (Figure 6B). So it can be inferred that both gene duplication and transcriptional regulation can lead to 1Bx7 overexpression, and their effects on 1Bx7 expression can be accumulated.

Endosperm is the storage tissue for starch and protein in cereal crops, which are the major sources of carbohydrates and proteins for humans. Improved yield and quality of crops by genetic modification has huge potential, and some significant achievements have already been accomplished [54]. Because continuous high expression of foreign genes in all tissues may cause detrimental effects in host plants [55], identification and application of strong endosperm-specific promoters will attract interest from breeders and biologists. In the current work, we identified a highly active *1Bx7^OE^* promoter that can enhance endosperm-specific gene expression at the transcriptional level, and it should be useful for wheat quality improvement by means of genetic transformation and molecular assisted breeding.

## Supporting Information

Figure S1 **PCR amplification of** ***1Bx*** **promoters by using 1Bx1007-F/R (A) and 1Bx2258-F/R (B) primer pairs.** Lane 1–4: Chinese Spring (*Pro-1Bx7*); Yunmai 33 (*Pro-1Bx7^OE^*); Yanzhan 1 (*Pro-1Bx14*); Atlas 66 (*Pro-1Bx13*); M is a DNA ladder. The PCR products were separated in 1.5% agarose gels.
(PDF)

Figure S2 **Alignment of four** ***1Bx*** **promoters (** ***Pro-1Bx7*** **,** ***Pro-1Bx7^OE^*** **,** ***Pro-1Bx13*** **and** ***Pro-1Bx14*** **).**
(PDF)

Figure S3 **Representative transient expression results of GUS driven by** ***Pro-1Bx*** **in wheat endosperms. (A)** ***Pro-1Bx13*** **; (B)** ***Pro-1Bx7*** **.**
(PDF)

Figure S4 **Southern blot analysis of transgenic rice lines with full-length** ***1Bx*** **promoters (A) or truncated** ***1Bx*** **promoters (B).** Genomic DNA was digested by *Bam*HI and detected by GUS gene probes.
(PDF)

Table S1 **Primers used in this study.**
(PDF)

Table S2 **HMW-GS compositions of six wheat accessions.**
(PDF)

Table S3 **Details of 12 known endosperm-specific cis-elements in** ***1Bx*** **promoters.**
(PDF)

Table S4 **Fourteen wheat cultivars harboring the 43 bp insertion in the** ***1Bx*** **promoter were identified by marker screening.**
(PDF)
