# Supplementary material for: Expression of Wheat High Molecular Weight Glutenin Subunit 1Bx Is Affected by Large Insertions and Deletions Located in the Upstream Flanking Sequences
Source: PLoS One. 2014 Aug 18;9(8):e105363. doi: 10.1371/journal.pone.0105363 (PMC4136844; doi:10.1371/journal.pone.0105363)
Supplement: Figure S1 — PCR amplification of 1Bx promoters by using 1Bx1007-F/R (A) and 1Bx2258-F/R (B) primer pairs. Lane 1–4: Chinese Spring (Pro-1Bx7); Yunmai 33 (Pro-1Bx7OE); Yanzhan 1 (Pro-1Bx14); Atlas 66 (Pro-1Bx13); M is a DNA ladder. The PCR products were separated in 1.5% agarose gels. (PDF) [file pone.0105363.s001.pdf]

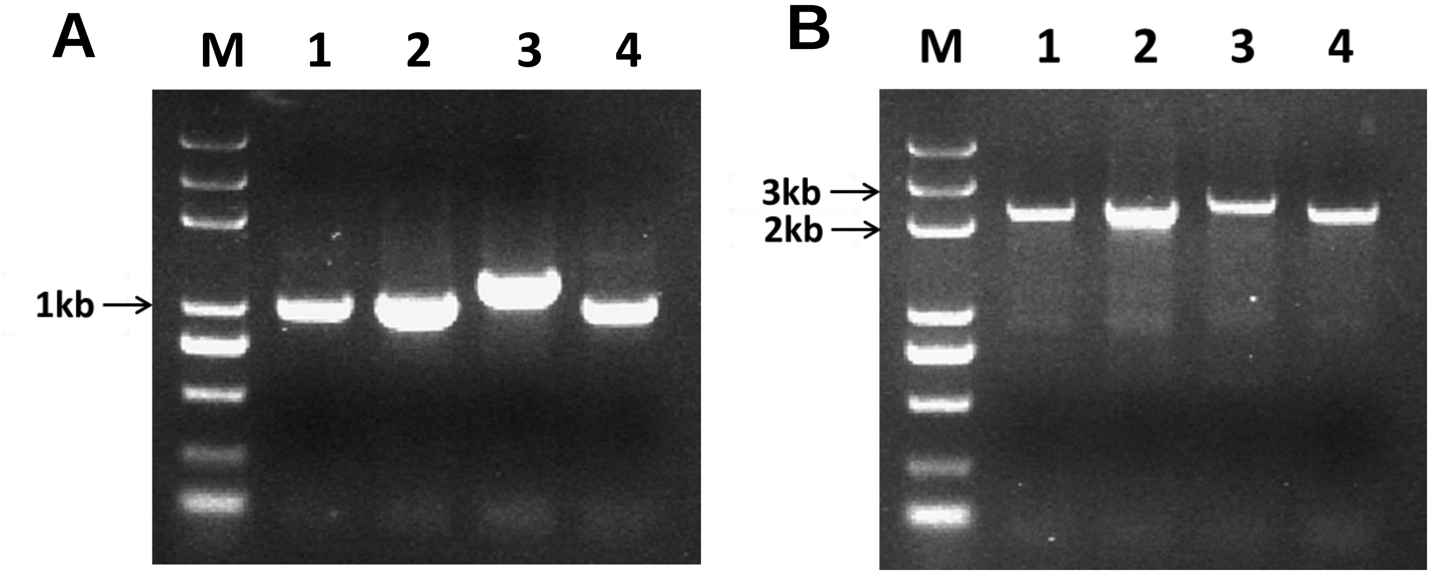

Figure S1. PCR amplification of *1Bx* promoters by using 1Bx1007-F/R (A) and 1Bx2258-F/R (B) primer pairs. Lane 1–4: Chinese Spring (*Pro-1Bx7*); Yunmai 33 (*Pro-1Bx7*^{OE}); Yanzhan 1 (*Pro-1Bx14*); Atlas 66 (*Pro-1Bx13*); M is a DNA ladder. The PCR products were separated in 1.5% agarose gels.
