# Supplementary material for: Expression of Wheat High Molecular Weight Glutenin Subunit 1Bx Is Affected by Large Insertions and Deletions Located in the Upstream Flanking Sequences
Source: PLoS One. 2014 Aug 18;9(8):e105363. doi: 10.1371/journal.pone.0105363 (PMC4136844; doi:10.1371/journal.pone.0105363)
Supplement: Figure S2 — Alignment of four 1Bx promoters ( Pro-1Bx7 , Pro-1Bx7OE , Pro-1Bx13 and Pro-1Bx14 ). (PDF) [file pone.0105363.s002.pdf]

```
Pro-1Bx7     TGATGTGCCCTTGCTTGATTTCAACATAGTGTGGTAAATTAAGTGGTACTAACAAAGACATATGCATTGTCAGTTTTTTCTTCTGCCAAACGAAATTTAAGCCTTTGTGTAAACACACA
Pro-1Bx7OE   TGATGTGCCCTTGCTTGATTTCAACATAGTGTGGTAAATTAAGTGGTACTAACAAAGACATATGCATTGTCAGTTTTTTCTTCTGCCAAACGAAATTTAAGCCTTTGTGTAAACACACA
Pro-1Bx13    TGATGTGCCCTTGCTTGATTTCAACATAGTGTGGTAAATTAAGTGGTACTAACAAAGACATATGCATTGTCAGTTTTTTCTTCTGCCAAACGAAATTTAAGCCTTTGTGTAAACACACA
Pro-1Bx14    TGATGTGCCCTTGCTTGATTTCAACATAGTGTGGTAAATTAAGTGGTACTAACAAAGACATATGCATTGTCAGTTTTTTCTTCTGCCAAACGAAATTTAAGCCTTTGTGTAAACACACA

Pro-1Bx7     GAGACAACTACAAACAACATCTCTGAAGAATTTAGCCAAATACATTGTAAACTATCTTGGGATATGTGCAAGGTCTGCTGGTATCTCCTTGCATGACGTGATAGTAAAGTAGCTTCTTGA
Pro-1Bx7OE   GAGACAACTACAAACAACATCTCTGAAGAATTTAGCCAAATACATTGTAAACTATCTTGGGATATGTGCAAGGTCTGCTGGTATCTCCTTGCATGACGTGATAGTAAAGTAGCTTCTTGA
Pro-1Bx13    GAGACAACTACAAACAACATCTCTGAAGAATTTAGCCAAATACATTGTAAACTATCATGGG.......CAAGGTCTGCTGGTATCTCCTTGCATGACGTGATAGTAAAGTAGCTTCTTGA
Pro-1Bx14    GAGACAACTACAAACAACATCTCTGAAGAATTTAGCCAAATATATTGTAAACTATCTTGGGATATGTGCAAGGTCTGCTGGTATCTCCTTGCATGACGTGATAGTAAAGTAGCTTCTTGA

Pro-1Bx7     ATAAGATTTGAAAAAATTCTCCTTGCTGGGTCAATAATTGTTTTTTTCTCTCTCTCAAGGAGTCCTCTCATTCCATTCTTTTAATTCATTATGCTCCCTAATTTTGGAGCTCTCCCATCCA
Pro-1Bx7OE   ATAAGATTTGAAAAAATTCTCCTTGCTGGGTCAATAATTGTTTTTTTCTCTCTCTCAAGGAGTCCTCTCATTCCATTCTTTTAATTCATTATGCTCCCTAATTTTGGAGCTCTCCCATCCA
Pro-1Bx13    ATAAGATTTGAAAAAATTCTCCTTGCTGGGTCAATAATTGTTTTTTT.TCTCTCTCAAGGAGTCCTCTCATTCCATTCTTTTAATTCATTATGCTCCCTAATTTTGGAGCTCTCCCATCCA
Pro-1Bx14    ATAAGATTTGAAAAAATTCTCCTTGCTGGGTCAATAATTGTTTTTTTTTCTCTCTCAAGGAGTCCTCTCATTCCATTCTTTTAATTCATTATGCTCCCTAATTTTGGAGCTCTCCCATCCA

Pro-1Bx7     ATTGAAATATTCAATTTTAGATTTGGTTCACAATTTTGATCGGGTCGATGGTATCAATCCCGCGCATCCTCTCACCATAAAA.AAAAGAGTCAATATATGATATTGTAGCATTAATACA
Pro-1Bx7OE   ATTGAAATATTCAATTTTAGATTTGGTTCACAATTTTGATCGGGTCGATGGTATCAATCCCGCGCATCCTCTCACCATAAAA.AAAAGAGTCAATATATGATATTGTAGCATTAATACA
Pro-1Bx13    ATTGAAATATTCAATTTTAGATTTGGTTCACAATTTTGATCGGGTCGATGGTATCAATCCTGCGCATCCTCTCACCATAAAA.AAAAGAGTCAATATATGATATTGTAGCATTAATACA
Pro-1Bx14    ATTGAAATATTCAATTTTAGATTTGGTTCACAATTTTGATCGGGTCGATGGTATCAATCCCTCGCATCCTCTCACCATAAAAGAAAAAAGTCAATATATGATATTGTAGCATTAATACA

Pro-1Bx7     TACATGTAGCCACCGACATGGAAAACGTCCATGCATAAGTATGGTTGATTAATGAAAAACTCATAATCACACTGCATTTTTATAAATAAAAAGAAAATACACTTCATCATCTTCCTCACA
Pro-1Bx7OE   TACATGTAGCCACCGACATGGAAAACGTCCATGCATAAGTATGGTTGATTAATGAAAAACTCATAATCACACTGCATTTTTATAAATAAAAAGAAAATACACTTCATCATCTTCCTCACA
Pro-1Bx13    TACATGTAGCCACCGACATGGAAAACGTCCATGCATAAGTATGGTTGATTAATGAAAAACTCATAATCACACTGCATTTTTATAAATAAAAAGAAAATACACTTCATCATCTTCCTCACA
Pro-1Bx14    TACATGTAGCCACCGACATGGAAAACGTCCATGCATAAGTATGGTTGATTAATGAAAAACTCATAATCACACTGCATTTTTATAAATAAAAAGAAAATACACTTCATCATCTTCCTCACA

Pro-1Bx7     CACCAAGCCAAATACTCCATCTGTTTCTTTGACCAAGTTCACAACCAAAAACATCAATATCCATAATACTAAATAAATAAAATATGAAAATTCGTTTCATGCTTAATCCAATGATATCGA
Pro-1Bx7OE   CACCAAGCCAAATACTCCATCTGTTTCTTTGACCAAGTTCACAACCAAAAACATCAATATCCATAATACTAAATAAATAAAATATGAAAATTCGTTTCATGCTTAATCCAATGATATCGA
Pro-1Bx13    CACCAAGCCAAATACTCCATCTGTTTCTTTGACCAAGTTCACAACCAAAAACATCAATATCCATAATACTAAATAAATAAAATATGAAAATTCGTTTCATGCTTAATCCAATGATATCGA
Pro-1Bx14    CACCAAGCCAAATACTCCATCTGTTTCTTTGACCAAGTTCACAACCAAAAACATCAATATCCATAATACTAAATAAATAAAATATGAAAATTCGTTTCATGCTTAATCCAATGATATCGA

Pro-1Bx7     CTTGATATTATGGATATTTATGTATTTCTCTACAAATTTGATCAAACTTTAAAAGGTTTAACTTCTCAAAAAAAAAAACTAATACACTTTATATTTTAAAACAGAGAGAGTAGTTTTTT
Pro-1Bx7OE   CTTGATATTATGGATATTTATGTATTTCTCTACAAATTTGATCAAACTTTAAAAGGTTTAACTTCTCAAAAAAAAAA.CTAATACACCTTATATTTTAAAACAGAGAGAGTAGTTTTTT
Pro-1Bx13    CTTGATATTATGGATATTTATGTATTTCTCTACAAATTTGATCAAACTTTAAAAGGTTTAACTTCTCAAAAAAAA...CTAATACACCTTATATTTTAAAACAGAGAGAGTAGTTTTTT
Pro-1Bx14    CTTGATATTATGGATATTTATGTATTTCTCTACAAATTTGATCAAACTTTAAAAGGTTTAACTTCTCAAAAAAAA...CTAATACACCTTATATTTTAAAACAGAGAGAGTAGTTTTTT

Pro-1Bx7     TTACCCAAACCCCAACTGCCCAGCAAAGCGCGCTCAACTCTTCTAGTCTAAATAACTAGCATCCACTAACACATTTCTCCCGACATGCAAGCACGTCACCTATGAAAATGCCCACCTCAA
Pro-1Bx7OE   TTACCCAAACCCCAACTGCCCAGCAAAGCGCGCTCAACTCTTCTAGTCTAAATAACTAGCATCCACTAACACATTTCTCCCGACATGCAAGCACGTCACCTATGAAAATGCCCACCTCAA
Pro-1Bx13    TTACCCAAACCCCAACTGCCCAGCAAAGCGCGCCCAACTCTTCTAGTCTAAATAACTAGCATCCACTAACACATTTCTCCCGACATGCAAGCACGTCACCTATGAAAATGCCCACCTCAA
Pro-1Bx14    T.ACCCAAACCCCAACTGCCCAGCAAAGCGCGCCCAACTCTTCTAGTCTAAATAACTAGCATCCACTAACACATTTCTCCCGACATGCAAGCACGTCACCTATGAAAATGCCCACCTCAA

Pro-1Bx7     TATGCAACCATGCATAGAAGAAAGCTCACCTCAGCATGCAAACATGCAGCATAATTTCCATTTTACTTGGCTATTTATGTTTGATAAATATTTCACAAATATACAATAATCAAAAACAA
Pro-1Bx7OE   TATGCAACCATGCATAGAAGAAAGCTCACCTCAGCATGCAAACATGCAGCATAATTTCCATTTTACTTGGCTATTTATGTTTGATAAATATTTCACAAATATACAATAATCAAAAACAA
Pro-1Bx13    TATGCAACCATGCATAGAAGAAAGCTCACCTCAGCATGCAAACATGCAGCATAATTTCCATTTTACTTGGCTATTTATGTTTGATAAATATTTCACAAATATACAATAATCAAAAACAA
Pro-1Bx14    TATGCAACCATGCATAGAAGAAAGCTCACCTCAGCATGCAAACATGCAGCATAATTTCCATTTTACTTGGCTATTTATGTTTGATAAATATTTCACAAATATACAATAATCAAAAACAA

Pro-1Bx7     AAATTATATGTGTTTTTAGTTTTAGTTCTCATATCCAAATATACATGTTTCATACAACCAAATCTCATTTAAATATATTGTAAAATATTCCGGCAACAACTTGTGGGG...........
Pro-1Bx7OE   AAATTATATGTGTTTTTAGTTTTAGTTCTCATATCCAAATATACATGTTTCATACAACCAAATCTCATTTAAATATATTGTAAAATATTCCGGCAACAACTTGTGGGGGCCTTAAATATA
Pro-1Bx13    AAATTATACGTGTTTTTAGTTTTAGTTCTCATATCCAAATATACATGTTTCATACAACCAAATCTCATTTAAATATATTGTAAAATATTCCGGCAACAACTTGTGGGG...........
Pro-1Bx14    AAATTATATGTGTTTTTAGTTTTAGTTCTCATATCCAAATATACATGTTTCATACAACCAAATCTCATTTAAATATATTATAAAATATTCCAGCAACAACTTGTGGGG...........

Pro-1Bx7     ..............................TACATCTAGTTACAGTGGAATATTAGTGATGGCGTGACCAAGCGATAAGGCCAACGAGAGAAGAAGTGCGTCGTCTATGGAGGCCAGGC
Pro-1Bx7OE   TTGTAAAATATTCCGGCAACAACTTGTGGGTACATCTAGTTACAGTGGAATATTAGTGATGGCGTGACCAAGCGATAAGGCCAACGAGAGAAGAAGTGCGTCGTCTATGGAGGCCAGGC
Pro-1Bx13    ..............................TACATCTAGTTACAGTGGAATATTAGTGATGGCGTGAGCAAGCGATAAGGCCAACGAGAGAAGAAGTGCGTCGTCTATGGAGGCCAGGC
Pro-1Bx14    ..............................TACATCTAGTTACAGTGGAATATTAGTGATGGCGTGATCAAGCGATAAGGCCAACGAGAGAAGAAGTGCGTCGTCTATGGAGGCCAGGC
```

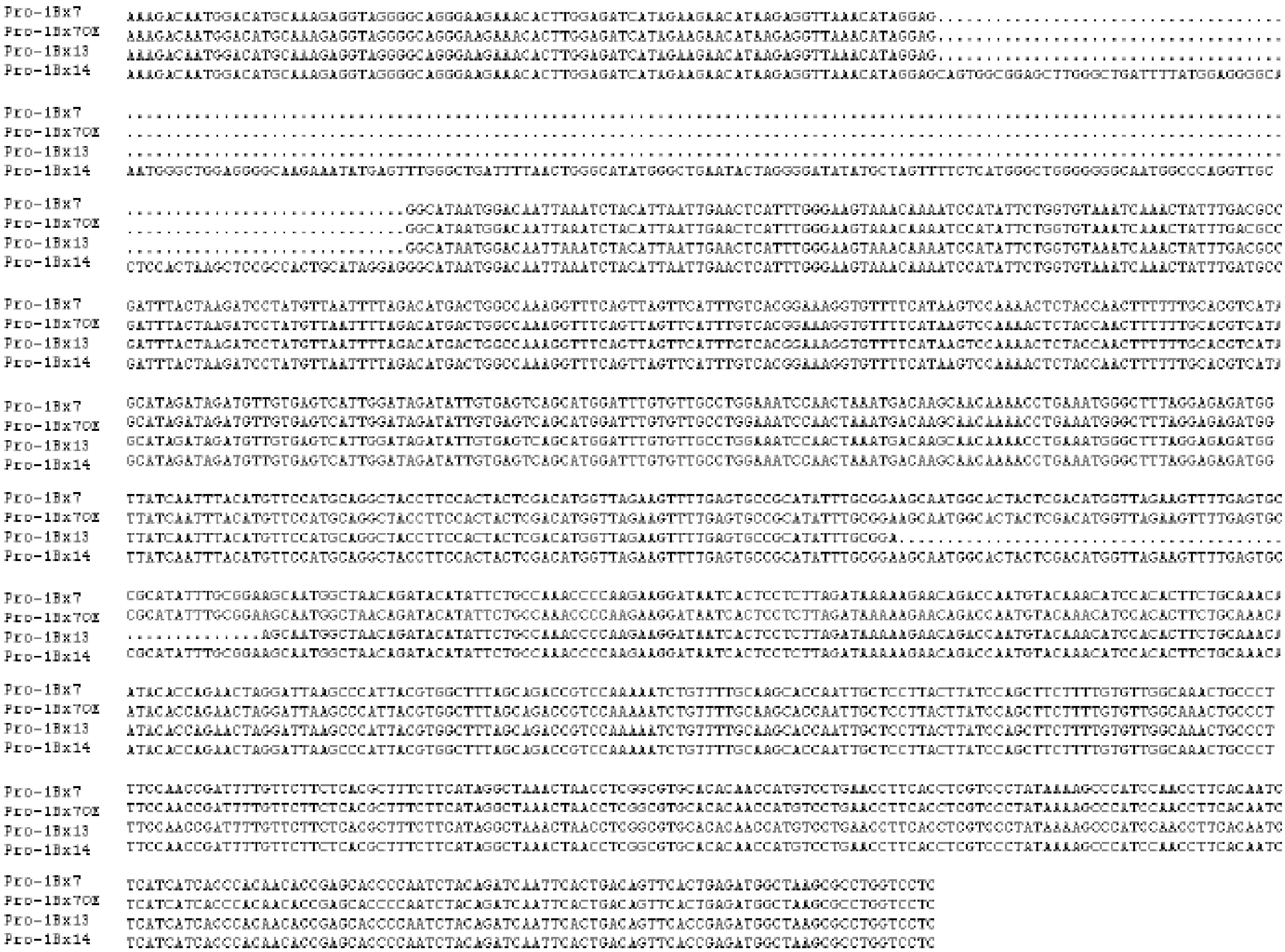

Figure S2. Alignment of four *1Bx* promoters (*Pro-1Bx7*, *Pro-1Bx7^{OE}*, *Pro-1Bx13* and *Pro-1Bx14*).
