# Supplementary material for: Expression of Wheat High Molecular Weight Glutenin Subunit 1Bx Is Affected by Large Insertions and Deletions Located in the Upstream Flanking Sequences
Source: PLoS One. 2014 Aug 18;9(8):e105363. doi: 10.1371/journal.pone.0105363 (PMC4136844; doi:10.1371/journal.pone.0105363)
Supplement: Figure S3 — Representative transient expression results of GUS driven by Pro-1Bx in wheat endosperms. (A) Pro-1Bx13 ; (B) Pro-1Bx7 . (PDF) [file pone.0105363.s003.pdf]

A B

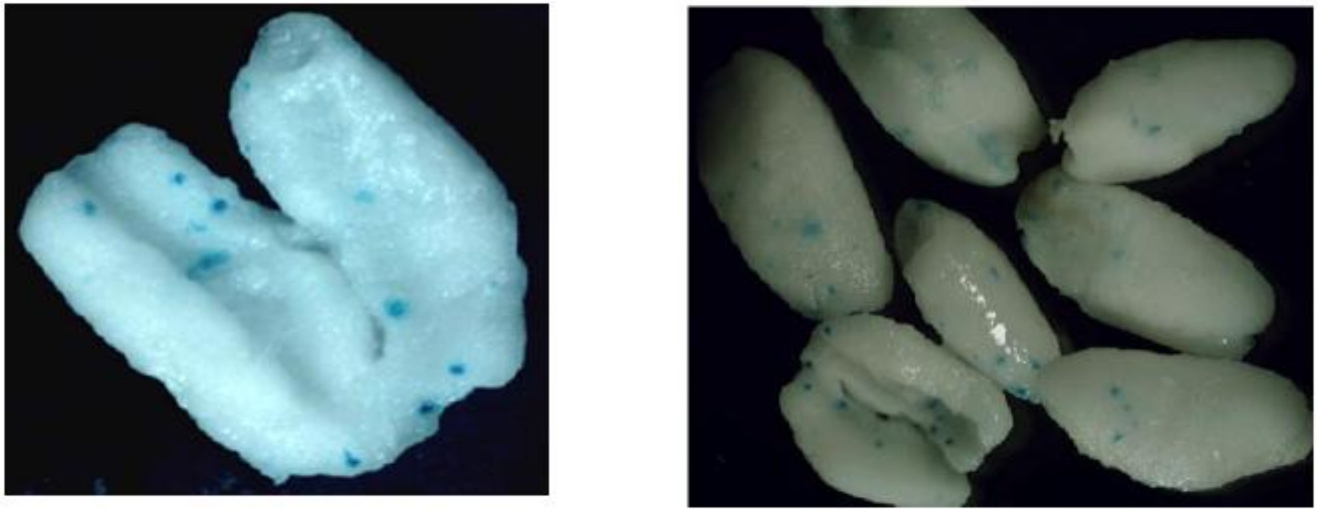

Figure S3. Representative transient expression results of GUS driven by *Pro-1Bx* in wheat endosperms. (A) *Pro-1Bx13*; (B) *Pro-1Bx7.*
