# Supplementary material for: Expression of Wheat High Molecular Weight Glutenin Subunit 1Bx Is Affected by Large Insertions and Deletions Located in the Upstream Flanking Sequences
Source: PLoS One. 2014 Aug 18;9(8):e105363. doi: 10.1371/journal.pone.0105363 (PMC4136844; doi:10.1371/journal.pone.0105363)
Supplement: Figure S4 — Southern blot analysis of transgenic rice lines with full-length 1Bx promoters (A) or truncated 1Bx promoters (B). Genomic DNA was digested by BamHI and detected by GUS gene probes. (PDF) [file pone.0105363.s004.pdf]

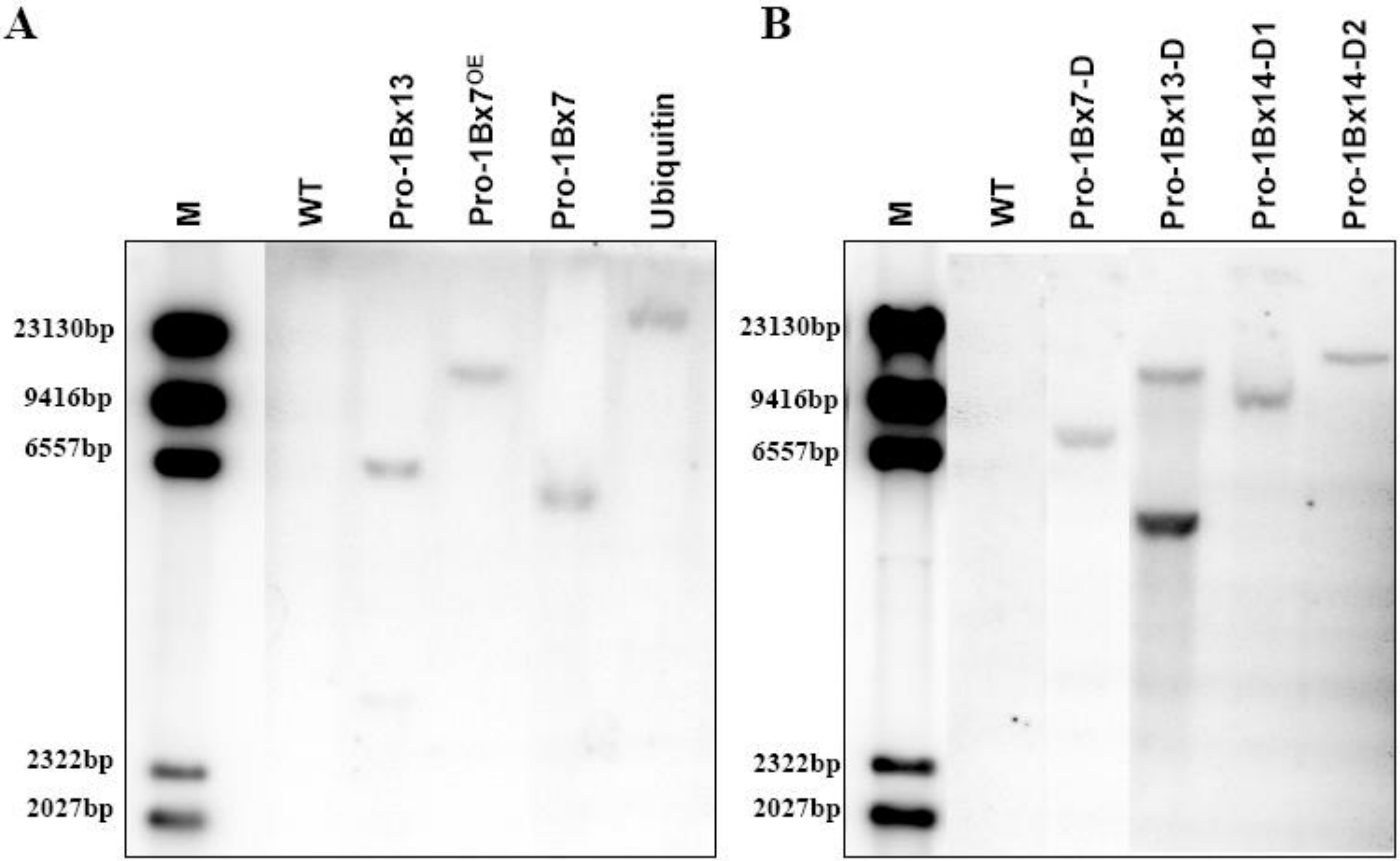

Figure S4. Southern blot analysis of transgenic rice lines with full-length *1Bx* promoters (A) or truncated *1Bx* promoters (B). Genomic DNA was digested by *Bam*HI and detected by GUS gene probes.
