# Supplementary material for: Expression of Wheat High Molecular Weight Glutenin Subunit 1Bx Is Affected by Large Insertions and Deletions Located in the Upstream Flanking Sequences
Source: PLoS One. 2014 Aug 18;9(8):e105363. doi: 10.1371/journal.pone.0105363 (PMC4136844; doi:10.1371/journal.pone.0105363)
Supplement: Table S1 — Primers used in this study. (PDF) [file pone.0105363.s005.pdf]

Table S1. Primers used in this study.

NO.	Primer names	Primer sequences
1	1Bx2258 F	5'-TGATGTGCCCTTGCTTGATTT-3'
2	1Bx2258 R	5'-AAAGAGGACCAGGCGCTTAGC-3'
3	1Bx1007 F	5'- CCAACAAGAGAAGAAGTGCGTCGTC-3'
4	1Bx1007 R	5'-GCTTAGCCATCTCGGTGAACTGTC-3'
5	1Bx2258/HindIII F	5'-TATACAAGCTTCCCTTGCTTGATTT-3'
6	1Bx2258/ BamHI R (for1Bx7/1Bx7^{OE})	5'-TTCGGGATCCTCAGTGAACTGTCA-3'
7	1Bx2258/BamHI R(for 1Bx13)	5'-TTCGGGATCCTCGGTGAACTGTCA-3'
8	1Bx1007/BamHI R	5'-TTCGGGATCCTCGGTGAACTGTCAGTGA-3'
9	1Bx1007/ SphI F	5'-ACATGCATGCCAACAAGAGAAGAAGTGC-3'
10	1Bx845/ SphI F	5'-ACATGCATGCGGGCATAATGGACAA-3'
11	GUS-F	5'-GCGGCTATACGCCATTTGAA-3'
12	GUS-R	5'-GTGAGCGTCGCAGAACATTACA-3'
13	Real-GAPDH F	5'-CGACCCGTTCATCACCACCGAC-3'
14	Real-GAPDH R	5'-AGCTAGCAGCCCTTCCACCTCTCCA-3'
15	Real-GUSF1115	5'-CAACGGGGAAACTCAGCAAG-3'
16	Real-GUSR1330	5'-TGAGCGTCGCAGAACATTACA-3'
17	43F	5'-TTTTACCCAAACCCCAACTG-3'
18	43R	5'-CCTCCATAGACGACGCACTT-3'

Note: No.1-4 were the common primers for cloning the 5' flanking sequence of ***1Bx*** genes, the number 2258 and 1007 indicate the cloned product length. No. 5-10 were the primers for constructing the GUS vectors. No.11-12 were the primers for probes labeled in Southern blotting. No.13-16 were the primers of internal control and *GUS* gene used in qRT-PCR. No.17-18 were the primers for the functional molecular maker in population screening.
