# Supplementary material for: Expression of Wheat High Molecular Weight Glutenin Subunit 1Bx Is Affected by Large Insertions and Deletions Located in the Upstream Flanking Sequences
Source: PLoS One. 2014 Aug 18;9(8):e105363. doi: 10.1371/journal.pone.0105363 (PMC4136844; doi:10.1371/journal.pone.0105363)
Supplement: Table S2 — HMW-GS compositions of six wheat accessions. (PDF) [file pone.0105363.s006.pdf]

Table S2. HMW-GS compositions of 6 wheat accessions

NO.	Cultivars and Accessions	HMW-GS Compositions		
		Glu-A1	Glu-B1	Glu-D1
1	Xiaoyan 54	1	14+15	2+12
2	Atlas 66	2*	13+16	2+12
3	Chinese Spring	N	7+8	2+12
4	Yunmai 33	1	7^{OE}+8	2+12
5	Jimai 20	1	13+16	5'+12
6	Yanzhan 1	N	14+15	5'+12
