# Supplementary material for: Expression of Wheat High Molecular Weight Glutenin Subunit 1Bx Is Affected by Large Insertions and Deletions Located in the Upstream Flanking Sequences
Source: PLoS One. 2014 Aug 18;9(8):e105363. doi: 10.1371/journal.pone.0105363 (PMC4136844; doi:10.1371/journal.pone.0105363)
Supplement: Table S3 — Details of 12 known endosperm-specific cis-elements in 1Bx promoters. (PDF) [file pone.0105363.s007.pdf]

Table S3. Details of 12 known endosperm-specific *cis*-elements in *1Bx* promoters.

Name	1Bx7	$1Bx7^{OE}$	1Bx13	1Bx14	Source
**DOF recognition sites**					
P-box1(TGCAAAG)	-994	-944	-890	-1129	Sugiyama et al., 1985
P-box2(TGCAAAC)	-1239,-311	-1282,-270	-1184, -311	-1424,-311	Norre et al., 2002
P-box 3(TGCAAG)	-2042,-1310,<br>-241,-2020	-2084,-1353,<br>-241,-2062	-1256,-241,<br>-1963	-2224,-1495,<br>-241, -2203	Thomas and Flavell, 1990
**bZIP recognition sites**					
Skn-1 like motif(GTCAT)	-669,-644,<br>-751,-586	-669,-644,<br>-710,-585	-615,-590,<br>-698,-532	-669,-644,<br>-752,-586	Blackwell et al., 1994
GCN4-like(GTGAGTCAT)	-648	-648	-594	-648	Albani et al., 1997
G-box like(TGACGT)	-1302,-670	-1318,-670	-1248,-617	-1487,-670	Menkens et al., 1995
**MYB recognition sites**					
AACA/TA motif 1(TAACAA)	-2179	-2220	-2114,	-2361	Diaz et al., 2002
AACA/TA motif 2(AACAAA)	-2178,-825,<br>-577,-171	-2219,-825,<br>-577,-171	-2113,-771,<br>-523,-171	-2360,-825,<br>-577,-171	Diaz et al., 2002
MYB1 AT core(AAACCA)	-547	-546	-493	-546	Abe et al., 1997
**VP1 recognition site**					
RY core site(CATGCA)	-1726,-1312,<br>-1267,-1241,<br>-1233,-946,-525	-1768,-1356<br>-1310,-1284,<br>-1276,-946,-525	-1669,-1258,<br>-1213,-1187,<br>-1179,-891,-470	-1497, -1452,-<br>1426, -1418,<br>-1131, -907,-525,	Suzuki et al., 1997
**Basal promoter sites**					
TATA like motif(TATAA)	-91	-91	-91	-91	Bernard et al., 2010
CCAAT Box(CCAAT)	-36,-233	-36,-233	-36,-233	-36,-233	Albani and Robert, 1995
