# Supplementary material for: Expression of Wheat High Molecular Weight Glutenin Subunit 1Bx Is Affected by Large Insertions and Deletions Located in the Upstream Flanking Sequences
Source: PLoS One. 2014 Aug 18;9(8):e105363. doi: 10.1371/journal.pone.0105363 (PMC4136844; doi:10.1371/journal.pone.0105363)
Supplement: Table S4 — Fourteen wheat cultivars harboring the 43 bp insertion in the 1Bx promoter were identified by marker screening. (PDF) [file pone.0105363.s008.pdf]

Table S4. Fourteen wheat cultivars harboring the 43 bp insertion in the *1Bx* promoter were identified by marker screening.

	Names of cultivars	Times of breeding	Origins	Types of 1Bx	Gene duplication[#]
Chinese cultivars	Enmai 4	1980	China (Hubei)	1Bx7	N
	Chuanmai 10	1968	China (Sichuan)	1Bx7	N
	Yunmai 33	1982	China (Yunnan)	1Bx7	Y
European cultivars	Pane-247	1960	Spanish	1Bx7	N
	Tadepi	1949	France	1Bx7	N
	GK Bence	1985	Hungary	1Bx6	N
	Frassinetto 405	1927	Italy	1Bx7	N
	Funo	1944	Italy	1Bx14	N
	Funone	1955	Italy	1Bx7	N
	Lario	1969	Italy	1Bx7	N
	Pegaso	1987	Italy	1Bx7	N
	Komorowska-pol	1951	Poland	1Bx6	N
	Ostka popularna	1953	Poland	1Bx7	N
	Amarelo de barba branca	1927	Portugal	1Bx14	N

Note: We have screened 505 Chinese and 160 European cultivars by using new developed functional maker based on 43 bp insertion and found this insertion existing in *1Bx* promoters of 14 cultivars in all. There are three types of 1Bx subunit, 1Bx7 (including $1Bx7^{OE}$), 1Bx14 and 1Bx6 respectively.

Gene duplication was detected by using molecular markers developed by Ragupathy et al. (2008). Y: Yes; N: No. Only Yumai 33 has *1Bx7* gene duplication based on the markers.
